# Homozygous CDA*3 is a major cause of life-threatening toxicities in gemcitabine-treated Japanese cancer patients

**DOI:** 10.1038/sj.bjc.6604971

**Published:** 2009-03-17

**Authors:** H Ueno, N Kaniwa, T Okusaka, M Ikeda, C Morizane, S Kondo, E Sugiyama, S R Kim, R Hasegawa, Y Saito, T Yoshida, N Saijo, J Sawada

**Affiliations:** 1 Hepatobiliary and Pancreatic Oncology Division, National Cancer Center Hospital, 5-1-1 Tsukiji, Chuo-ku, Tokyo 104-0045, Japan; 2 Project Team for Pharmacogenetics, National Institute of Health Sciences, 1-18-1 Kamiyoga, Setagaya-ku, Tokyo 158-8501, Japan; 3 Division of Medicinal Safety Sciences, National Institute of Health Sciences, 1-18-1 Kamiyoga, Setagaya-ku, Tokyo 158-8501, Japan; 4 Division of Functional Biochemistry and Genomics, National Institute of Health Sciences, 1-18-1 Kamiyoga, Setagaya-ku, Tokyo 158-8501, Japan; 5 Genetics Division, National Cancer Center Research Institute, 5-1-1 Tsukiji, Chuo-ku, Tokyo 104-0045, Japan; 6 National Cancer Center Hospital East, 6-5-1 Kashiwanoha, Kashiwa-shi, Chiba 277-0882, Japan

**Keywords:** gemcitabine, toxicity, *CDA*208G>A, pancreatic cancer, pharmacogenomics, polymorphism

## Abstract

Among 242 Japanese pancreatic cancer patients, three patients (1.2%) encountered life-threatening toxicities, including myelosuppression, after gemcitabine-based chemotherapies. Two of them carried homozygous CDA*3 (*CDA*208G>A [Ala70Thr]), and showed extremely low plasma cytidine deaminase activity and gemcitabine clearance. Our results suggest that homozygous *3 is a major factor causing gemcitabine-mediated severe adverse reactions among the Japanese population.

Gemcitabine (2′,2′-difluorodeoxycytidine) is a nucleoside anticancer drug for various solid tumours (Noble and Goa, 1997). Gemcitabine exerts its cytotoxic effect through phosphorylation by nucleotide kinases, including the deoxycytidine kinase (DCK), whereas most of the administered gemcitabine is rapidly degraded by cytidine deaminase (CDA) into its inactive metabolite, 2′,2′-difluorodeoxyuridine (Plunkett *et al*, 1995). Various genetic variations have recently been reported in human *DCK* and *CDA* genes (Ueno *et al*, 2007).

Our earlier prospective pharmacogenetic study using 256 Japanese cancer patients treated with gemcitabine-based chemotherapies revealed that one of the *CDA* single-nucleotide polymorphisms (SNPs), CDA*3 (*CDA*208G>A [Ala70Thr], rs60369023), showed significant associations with reduced CDA activity, reduced gemcitabine clearance, increased gemcitabine area under the concentration–time curve (AUC), and an increased incidence of severe neutropaenia (Sugiyama *et al*, 2007). Most notably, one patient who had developed life-threatening toxicities, including severe myelosuppression, was found to be homozygous for CDA*3 (CDA*3/*3), and excessive exposure to gemcitabine was considered responsible for the severe toxicities (Yonemori *et al*, 2005; Sugiyama *et al*, 2007).

Owing to a low allele frequency of CDA*3 (3.7% in the Japanese population), only one homozygous patient was found in the earlier study, necessitating further examination. For this purpose, we have carefully monitored toxicities in gemcitabine-treated patients in the National Cancer Center Hospital for 4.5 years. Three patients with life-threatening adverse reactions, including serious myelosuppression, were identified, and their *CDA* genotypes, plasma CDA activities, and pharmacokinetic parameters (when available) were determined and compared with those of the earlier cases.

## Patients and methods

The ethics committees of the National Cancer Center and the National Institute of Health Sciences approved this study. Written informed consent was obtained from each participant. Of 176 and 66 pancreatic cancer patients who received gemcitabine monotherapy and gemcitabine-based combination chemotherapies, respectively, at the National Cancer Center Hospital between 1 September 2003 and 31 March 2008, three showed severe and prolonged myelosuppression with other complications. Characteristics of the three patients designated A, B, and C are summarised in Table 1. All these patients received a 30 min intravenous gemcitabine infusion at an initiation dose of 1000 mg m^−2^. Patient A initially received gemcitabine and S-1 combination therapy, whereas patients B and C were given gemcitabine alone.

Measurement of plasma CDA activity towards gemcitabine and genotyping of *CDA* and *DCK* were carried out in the three patients as reported earlier (Sugiyama *et al*, 2007; Kim *et al*, 2008). After recovery from severe adverse reactions, chemotherapy was resumed in two patients: patient A received gemcitabine monotherapy instead of gemcitabine plus S-1, whereas the gemcitabine dose was reduced in patient C. Gemcitabine was not resumed in patient B because of disease progression. Pharmacokinetics were carried out when 1000 and 450 mg m^−2^ of gemcitabine were administered to patients A and C, respectively. Blood sampling schedule and the measurement method of gemcitabine in plasma were reported earlier (Sugiyama *et al*, 2007). Pharmacokinetic parameters were estimated using WinNonlin ver 4.01 (Pharsight Corporation, Mountain View, CA, USA).

## Results

Although 30 patients (about 12.4%) among the 242 developed grade 4 neutropaenia, the toxicity was transient and required no supportive treatment in most patients except in patients A, B, and C. Pretreatment organ functions, including bone marrow, renal, and hepatic functions, were preserved in the three patients (Table 1). Observed toxicities in the patients are summarised in Table 2. The serious haematotoxicities requiring intensive supportive treatments during hospitalisation were recognised in these patients: patient A was treated with antibiotics because of febrile neutropaenia, patient B received a platelet transfusion, and patient C received a red blood cell transfusion, a platelet transfusion, and a granulocyte colony-stimulating factor. Both the neutrophil and platelet nadir appeared at approximately day 15 of the first course of treatment in patients A and B, whereas in patient C, the nadir occurred on day 15 of the second course of treatment that was resumed after reducing the dose of gemcitabine. The symptomatic non-haematologic toxicities shown in Table 2 appeared before severe myelosuppression.

**Figure 1:**
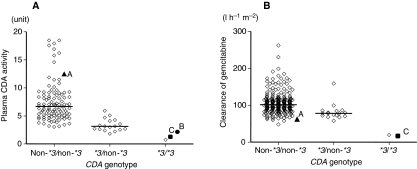
Effects of CDA*3 on (**A**) plasma CDA activity towards gemcitabine and (**B**) gemcitabine clearance. The data obtained earlier (Sugiyama *et al*, 2007) are expressed as open diamonds, and those obtained in this study are as closed symbols. A, B, and C represent patients A, B, and C, respectively. Lines represent median values for non-*3/non-*3 patients and *3/non-*3 patients.

Patients B and C were found to be CDA*3/*3, whereas patient A did not have CDA*3 (Table 2). No SNPs of *DCK,* including *DCK*364C>T (Pro122Ser), which were reported to have reduced enzymatic activity (Lamba *et al*, 2007), were identified in our three patients. Plasma CDA activities of patients A, B, and C were compared with those of 121 patients in our earlier study (Sugiyama *et al*, 2007) (Figure 1A). Patient A without CDA*3 showed relatively high plasma CDA activity, whereas plasma CDA activities in the CDA*3/*3 patients (patients B and C) were comparably low to those in the earlier reported CDA*3/*3 patient.

Pharmacokinetic parameters of patients A and C were also shown in Table 2, and their gemcitabine clearances were compared with those of the earlier reported 250 patients (Sugiyama *et al*, 2007) (Figure 1B). Although the gemcitabine dose for patient C was low (450 mg m^−2^), her gemcitabine AUC was higher than the average value in the CDA*3-negative patients who were administered 1000 mg m^−2^ gemcitabine. When it was assumed that patient C received 1000 mg m^−2^ of gemcitabine, *C*_max_ and AUC values similar to those observed in the earlier reported CDA*3/*3 patient were obtained. The value of gemcitabine clearance in patient C (CDA*3/*3) was less than one-fifth of the median value obtained earlier from CDA*3-negative patients. The clearance observed in patient A, who did not have CDA*3, was within the range obtained earlier from patients without CDA*3 (Figures 1B).

## Discussion

We found two (patients B and C) of the three patients who experienced life-threatening adverse reactions to be homozygous for CDA*3 and to have an extremely low CDA activity. Taken together with our earlier observations (Yonemori *et al*, 2005; Sugiyama *et al*, 2007), these life-threatening adverse reactions appear to have been caused by reduced deamination activity of CDA because of CDA*3/*3 homozygosity. Sustained plasma gemcitabine elevations are most likely responsible for these severe adverse reactions. To date, we have had three CDA*3/*3 patients, including one reported earlier (Yonemori *et al*, 2005; Sugiyama *et al*, 2007), and all experienced life-threatening severe adverse reactions. As genotyping of *CDA* was not carried out in the remaining 239 patients, we may have overlooked patients with CDA*3/*3 who did not develop severe toxicities in this study. However, as the frequency of homozygous CDA*3 in the Japanese population was estimated to be 0.14% in an earlier study (Sugiyama *et al*, 2007), the possibility of missing any such patients would be very low. Thus, CDA*3/*3 is a potentially important biomarker for Japanese patients and for at least one African ethnic group (Fukunaga *et al*, 2004) for predicting severe gemcitabine-mediated adverse reactions including myelotoxicities.

We do not have sufficient pharmacokinetic data on CDA*3/*3 to determine the optimal dose for this fraction of the patient population. However, the clearance and AUC values for the earlier reported patient (Sugiyama *et al*, 2007) and for patient C (dose adjusted), and the final dose for patient C (270 mg m^−2^) suggest that a 75% reduction in the gemcitabine dose, at treatment initiation, may be appropriate for CDA*3/*3 patients.

Patient A was given combined chemotherapy with oral S-1 (Table 1). As she could not tolerate gemcitabine monotherapy at the standard dose as shown in Table 2, gemcitabine itself appears to have been responsible for the life-threatening toxicities in this patient. Her gemcitabine clearance was within the range observed in patients without CDA*3 (Sugiyama *et al*, 2007). Therefore, we concluded that there was no involvement of altered CDA activity in the severe neutropaenia experienced by patient A. Further investigation of gemcitabine pathway genotypes is needed to clarify factors contributing to this patient's adverse reactions.

In conclusion, in the Japanese population, CDA*3/*3 is a major cause of gemcitabine-mediated life-threatening adverse reactions including myelosuppression. A substantial gemcitabine dose reduction is necessary for patients who are homozygous for CDA*3.

## Figures and Tables

**Table 1:** Patient characteristics at baseline

	**Patient A**	**Patient B**	**Patient C**
Sex	Female	Male	Female
Age (years)	57	70	70
Performance status	1	2	1
Stage^a^	IV	IV	IV
Previous treatment	Surgery	None	None
Body surface area (m^2^)	1.3	1.9	1.1
			
*Laboratory data*			
Leukocyte (mm^−3^)	6300	9700	4300
Neutrophil (mm^−3^)	4200	6400	2700
Haemoglobin (g dl^−1^)	12.4	16.0	10.8
Platelet (mm^−3^)	116 000	163 000	185 000
Total bilirubin (mg dl^−1^)	0.6	1.4	0.8
ALT (IU l^−1^)	26	35	24
Creatinine (mg dl^−1^)	0.6	1.2	0.4
Initial regimen	Gemcitabine+S-1^b^	Gemcitabine alone	Gemcitabine alone

ALT=alanine aminotransferase.

a UICC sixth edition.

b An oral product containing tegafur, gimeracil, and oteracil potassium.

**Table 2:** Toxicities, treatment, genotype, and PK analysis

	**Patient A**		**Patient B**		**Patient C**	
	**Grade**	**Value**	**Grade**	**Value**	**Grade**	**Value**
*Haematologic toxicities* a						
Leukocyte (mm^−3^)	4	800	3	1100	3	1000
Neutrophil (mm^−3^)	4	200	4	300	4	100
Haemoglobin (g dl^−1^)	2	8.1	1	13.2	4	6.3
Platelet (mm^−3^)	3	26 000	4	10 000	3	28 000
						
*Non-haematologic toxicity* a						
Fatigue	2		3		2	
Anorexia	3		3		2	
Diarrhoea	3		0		1	
Stomatitis	3		0		0	
Rash	2		0		2	
Febrile neutropaenia	3		0		0	
						
*Treatment*						
Resumption of chemotherapy	Yes		No		Yes	
Total number of gemcitabine doses	10		2		10	
Final dose (mg m^−2^)	600		1000		270	
			
*Genotype*						
*CDA* haplotype^b^	*1a/*1j		*3a/*3a		*3a/*3a	
*DCK* haplotype^c^	*1a/*1a		*1a/*1a		*1a/*1a	
			
*PK analysis*						
Regimen at PK study	Gemcitabine alone				Gemcitabine alone	
Dose of gemcitabine (mg m^−2^)	1000				450	
*C*_max_ (mg l^−1^)	29.0		Not available		22.5 (49.7^d^)	
AUC (mg h l^−1^)	16.2				26.8 (59.0^d^)	
Clearance (l h^−1^ m^−2^)	61.8				16.6	
					

AUC=area under the concentration–time curve; *C*_max_=maximum plasma concentration; CDA=cytidine deaminase; DCK=deoxycytidine kinase; PK=pharmacokinetics.

a Toxicities were assessed according to the National Cancer Institute Common Terminology Criteria for Adverse Events version 3.0.

b *CDA* haplotype was reported earlier in Sugiyama *et al* (2007).

c *DCA* haplotype was reported earlier in Kim *et al* (2008).

d On the basis of the assumption that patient C received 1000 mg m^−2^ of gemcitabine.
